# Cascaded deep learning classifiers for computer-aided diagnosis of COVID-19 and pneumonia diseases in X-ray scans

**DOI:** 10.1007/s40747-020-00199-4

**Published:** 2020-09-22

**Authors:** Mohamed Esmail Karar, Ezz El-Din Hemdan, Marwa A. Shouman

**Affiliations:** 1 Department of Computer Engineering and Networks, College of Computing and Information Technology, Shaqra University, Shaqra, Saudi Arabia; 2 Department of Industrial Electronics and Control Engineering, Faculty of Electronic Engineering, Menoufia University, Minuf, 32952 Egypt; 3 Department of Computer Science and Engineering, Faculty of Electronic Engineering, Menoufia University, Minuf, 32952 Egypt

**Keywords:** Coronavirus outbreak, COVID-19, Biomedical image processing, Deep learning, Cascaded classifiers

## Abstract

Computer-aided diagnosis (CAD) systems are considered a powerful tool for physicians to support identification of the novel Coronavirus Disease 2019 (COVID-19) using medical imaging modalities. Therefore, this article proposes a new framework of cascaded deep learning classifiers to enhance the performance of these CAD systems for highly suspected COVID-19 and pneumonia diseases in X-ray images. Our proposed deep learning framework constitutes two major advancements as follows. First, complicated multi-label classification of X-ray images have been simplified using a series of binary classifiers for each tested case of the health status. That mimics the clinical situation to diagnose potential diseases for a patient. Second, the cascaded architecture of COVID-19 and pneumonia classifiers is flexible to use different fine-tuned deep learning models simultaneously, achieving the best performance of confirming infected cases. This study includes eleven pre-trained convolutional neural network models, such as Visual Geometry Group Network (VGG) and Residual Neural Network (ResNet). They have been successfully tested and evaluated on public X-ray image dataset for normal and three diseased cases. The results of proposed cascaded classifiers showed that VGG16, ResNet50V2, and Dense Neural Network (DenseNet169) models achieved the best detection accuracy of COVID-19, viral (Non-COVID-19) pneumonia, and bacterial pneumonia images, respectively. Furthermore, the performance of our cascaded deep learning classifiers is superior to other multi-label classification methods of COVID-19 and pneumonia diseases in previous studies. Therefore, the proposed deep learning framework presents a good option to be applied in the clinical routine to assist the diagnostic procedures of COVID-19 infection.

## Introduction

Coronavirus Disease 2019 (COVID-19) initiated a pandemic in December 2019 in the city of Wuhan, China, causing a Public Health Emergency of International Concern (PHEIC) [1]. The COVID-19 is named by the World Health Organization (WHO) as a novel infectious disease, and it belongs to Coronaviruses (CoV) and perilous viruses [2, 3]. It results in some cases a critical care respiratory condition such as Severe Acute Respiratory Syndrome (SARS-CoV), leading to failure in breathing and the death eventually. Recently, situation report no. 74 of the WHO announced that the risk assessment of COVID-19 is very high at the global level on 3 April 2020 [4, 5]. In addition, the total number of cases has become 972,303 confirmed COVID-19 patients and 50,322 deaths worldwide. Also, other common lung infections like viral and bacterial pneumonia lead to thousands of deaths every year [6]. These pneumonia diseases cause fungal infection of one or both sides of the lungs by the formation of pus and other liquids in the air sacs. Symptoms of the viral pneumonia occur gradually and are mild. But bacterial pneumonia is more severe, especially among children [7]. This type of pneumonia can affect many lobes of the lung.

The gold standard for diagnosing common pneumonia diseases and Coronaviruses is the real-time polymerase chain reaction (RT-PCR) assay of the sputum [8]. However, these RT-PCR tests showed high false-negative levels to confirm positive COVID-19 cases. Alternatively, radiological examinations using chest X-ray and computed tomography (CT) scans are now being used to identify the health status of infected patients including children and pregnant women [9, 10], regardless of potential side effects of ionizing radiation exposure. The CT imaging presents an effective method for screening, diagnosis, and progress assessment of patients with COVID-19 [11]. Nevertheless, clinical studies demonstrated that a positive chest X-ray may obviate the need for CT scans and reducing clinical burden on CT suites during this pandemic outbreak [12, 13]. The American College of Radiology (ACR) recommended the utilization of portable chest radiography to minimize the risk of Coronavirus infection, because the decontamination of CT rooms after scanning COVID-19 patients may cause interruption of this radiological service [14]. Also, chest CT screening requires high-dose exposure to scan patients and relatively expensive hospital bills out [15]. In contrast, conventional X-ray machines are always available and portable in hospitals and clinical centers to give a quick scan for the patients’ lungs as two-dimensional (2D) images. Therefore, the chest X-ray scans present the first tool for clinicians to confirm positive COVID-19 cases [10, 16]. In this paper, we focus only on enhancing the performance of using chest X-ray scans for confirming the patients with highly suspected COVID-19 or other pneumonia diseases, namely viral (Non-COVID-19) or bacterial infections.

However, X-ray images are still contrast limited because of low-exposure dose to the patients, leading to difficulties in diagnosing soft tissues or diseased areas in the patient’s thorax [15, 17]. Computer-aided diagnosis (CAD) systems present a practical solution to overcome these limitations of chest X-rays, and to assist radiologists to automatically detect potential diseases in low-contrast X-ray images [18, 19]. The CAD systems combined advanced components of computer technologies with recent image processing algorithms to perform interventional tasks, e.g. tumor segmentation and 3D visualization of vital organs [20, 21]. Now, artificial intelligence (AI) has been widely applied to advance the diagnostic performance of many CAD systems for various medical applications such as brain tumor classification or segmentation [22, 23], minimally invasive aortic valve implantation [17], and detecting pulmonary diseases [24, 25]. Recently, deep learning approaches become the most advanced methods in the field of AI. They can learn patterns and features from labeled (or annotated) data to be capable of automatically performing specific tasks based on the previous training, such as human sentiment classification [26] and computer vision applications in surgery [27]. Convolutional neural networks (CNNs) present a major branch of deep learning techniques in many applications of computer vision and sensitive medical applications in the last years [28]. The CNNs have been used to analyze single and multi-modal medical images in different applications of radiology, e.g. breast cancer classification and lung nodule detection [29, 30].

### Related work

Automated diagnosis of COVID-19 and chest diseases has been investigated using medical CT and X-ray imaging modalities. Recent studies [31, 32] demonstrated that chest CT images and deep learning models play an important role to identify and segment COVID-19 infections successfully. However, this study is focused only on the utilization of chest X-ray images as a first tool to detect positive COVID-19 patients and other pneumonia diseases, as presented in the following previous studies: Automatic classification of lung diseases in X-ray images was proposed for tuberculosis screening [33], detection of consolidation in case of increased lung density [34], and critical pneumonia diseases [35]. But identifying the infectious status of novel COVID-19 in chest X-rays is still a new emerging research topic and appeared in a few studies of peer-review published articles. Deep learning classifiers such as the pre-trained InceptionV3 model have been used to confirm the presence of COVID-19 infection [36, 37]. Adding other pneumonia diseases for the proposed CNNs models improved the classification accuracy above 90.0%, as presented in [38, 39]. Drop-weight-based Bayesian CNNs were used to verify a high correlation of the uncertainty with the prediction accuracy of COVID-19 in X-ray images [40]. Ozturk et al. [41] proposed DarkCovidNet model as a single and multi-label classifier for COVID-19 and pneumonia diseases in X-ray radiographs, based on the real-time object detection algorithm, namely You Only Look Once (YOLO). The DarkCovidNet model achieved accuracy of 98.08% for binary classification and 87.02% for multi-class classification task. A threefold deep learning method has been proposed to perform top-down binary classification if the patient is healthy or affected by a pulmonary disease involving COVID-19 case with accuracy of 97.0% [42]. Also, this proposed method can provide a visualization of infected areas in X-ray images based Visual Geometry Group (VGG16) model. Another deep CNN model, called CoroNet was proposed for multi-class classification of COVID-19 and pulmonary diseases [43]. It has been developed based on the architecture of pre-trained Xception model [44], resulting an overall accuracy of 89.6%. Shaban et al. [45] introduced a new detection strategy of positive COVID-19 patients based on a hybrid selection method of the best image features and an enhanced K-nearest neighbor (EKNN) classifier. The proposed COVID-19 detection strategy has been tested on the chest CT images only. Deep feature extractor of a CNN model and Bayesian optimization have been used for detecting COVID-19 infection in chest X-ray images [46]. The extracted features were exploited as input variables to the machine learning algorithms, such as support vector machine (SVM). The SVM classifier resulted an accuracy of 98.7% to verify positive COVID-19 patients.

### Contributions of this study

This study aims at proposing a new deep learning framework as a radiological tool to support clinicians for automated diagnosis of COVID-19 and pneumonia diseases using chest X-rays. Our preliminary work (COVIDX-Net) [36] introduced deep learning models to confirm only positive or negative COVID-19 cases. In this article, we present a new version of our deep learning framework to constitute the following advancements:

- Developing a new cascaded form of deep learning image classifiers for confirming COVID-19, viral and bacterial pneumonia diseases.
- Using multiple selective and reliable deep learning models to achieve the best classification performance of pulmonary diseases in X-ray images.
- Proposed architecture of cascaded classifiers based on deploying different deep learning models allows to obtain better performance than could be obtained from other multi-label classifiers in previous studies.
- Extensive tests and evaluation of eleven deep learning models have been conducted on a public X-ray dataset to verify the best performance of proposed classifiers for detecting COVID-19 and other pneumonia diseases.
- Demonstrating the feasibility of applying our final recommended classifiers to enhance image-guided diagnosis of COVID-19, viral pneumonia, and bacterial pneumonia infections during the pandemic time.

The remainder of this article is divided into the following sections. “Materials and methods” describes the workflow of our proposed deep learning framework to automatically detect COVID-19, pneumonia viral, and bacterial in 2D radiographic images. The next section presents the experimental results and evaluation of all tested image classifiers. The conclusions and outlook of this study are given in the last section.

## Materials and methods

### Dataset

In this study, we used the public dataset of X-ray images including positive COVID-19 and other pneumonia cases collected by Dr. Cohen at the University of Montreal [47]. The dataset contains 306 X-ray images with four classes as 79 normal cases, 69 positive COVID-19 images, 79 images for viral (Non-COVID-19) pneumonia, and 79 bacterial pneumonia cases. Table 1 illustrates the distribution of X-ray images for training, validation and testing of our proposed deep learning framework. The size of all tested images is ranging from 1088 × 688 to 2567 × 2190 pixels. The main features of positive COVID-19 in chest X-rays are ground-glass opacification (GGO) and occasional consolidation in the bilateral patchy areas [10].

**Table 1:** Distribution of chest X-ray dataset over training, validation and testing for all cases in this study

Patient case	Number of chest X-ray images			
	Training	Validation	Testing	Total
Normal	56	11	12	79
COVID-19	49	9	11	69
Viral (Non-COVID-19) pneumonia	56	11	12	79
Bacterial pneumonia	56	11	12	79

### Description of proposed cascaded image classifiers

**Fig. 1:**
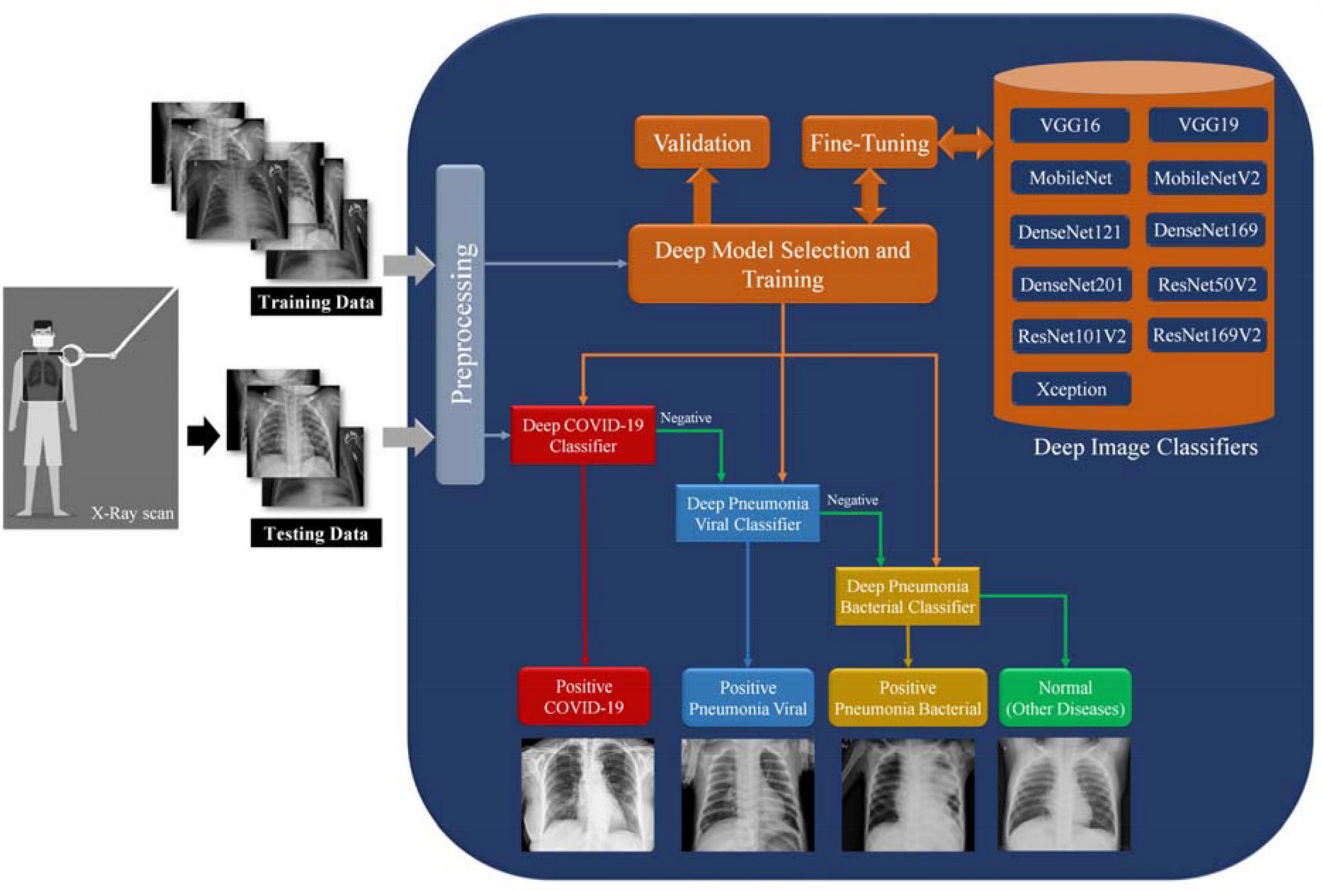
Workflow of proposed cascaded deep learning classifiers for confirming COVID-19, viral and bacterial pneumonia cases in chest X-rays

We proposed a new deep learning framework for detecting COVID-19, viral and bacterial pneumonia infections using chest X-ray scans. Figure 1 shows the workflow of our proposed framework includes eleven different pre-trained CNNs models, namely VGG16 [48], VGG19, Xception [44], Dense Convolutional Network (DenseNet-121) [49], DenseNet169, DenseNet201, Residual Neural Network (ResNet-50V2) [50], ResNet101V2, ResNet169V2, MobileNet [51], and MobileNetV2. In this study, the hyperparameter values of all deep learning models are fixed and listed in Table 2. As shown in Fig. 1, the proposed cascaded deep learning framework is composed of three main steps to perform automated diagnosis of COVID-19 and pneumonia diseases in X-ray scans as follows:

**Table 2:** Hyperparameter values of our proposed deep learning models in this study

Parameters	Value
Learning rate	0.001
Batch size	7.0
Number of epochs	50.0
Optimizer	Stochastic gradient descent (SGD)
Dropout	0.5
Activation function of the last classifier layer	Softmax

- Step #1: Preprocessing
  All images of tested X-ray dataset loaded and scaled to a constant size of 150 × 150 pixels for next processing steps in the pipeline of deep learning framework. Although the tested images are noise free, it is possible to apply any non-linear noise cancellation tool such as morphological filters, and the perceptual adaptation of the image (PAI), increasing the quality of input X-ray images [52, 53]. The labels of X-ray image data are binarized using one-hot encoding [54] to identify the positive cases of COVID-19 or viral pneumonia or bacterial pneumonia. Normal cases present here negative results of pathogenic diagnose, and the patient may have a pulmonary disease not defined in this study.
- Step #2: Training-selected model and validation
  Any pre-trained deep learning model is manually selected by the user for fine-tuning procedures. To overcome the limited data size of X-ray images, the top layers of all base models have been truncated and replaced with new fully connected layers to accomplish the fine-tuning task of all pre-trained deep learning models, as depicted in Fig. 1. The preprocessed X-ray dataset is 85–15 split because of the relatively small size of the dataset. Therefore, 15% of dataset approximately 10–12 images will be used for testing all classifiers. The rest of the images are initially used for randomly constructing training and validation sets. Then accuracy and loss metrics will be applied to evaluate the performance of both the training and validation of the trained model during 50 epochs for each selected deep image classifier.
- Step #3: Multi-stage chest disease classification
  Our proposed deep learning framework is a multi-stage classification process such that the priority is to confirm or “NOT” the positive COVID-19 cases. If the result of the first classifier is negative, the image data will be tested sequentially by the next two cascaded image classifiers of viral and bacterial pneumonia, as shown in Fig. 1. This study assumed that the patients have only a single disease if exists. At the end of the proposed workflow, the resulted performance of all tested models is quantitatively analyzed based on the classification evaluation metrics, as presented in the next section.

### Classification performance metrics

In this study, the classification performance of deep learning models was evaluated to quantify the results of diagnosed COVID-19 and two other pneumonia diseases in X-ray images. Different classification metrics have been applied as follows. Table 3 illustrates a basic 2 × 2 confusion matrix, which is estimated based on hypothesis testing and cross validation [55]. Comparing the true labels of tested images and predicted results of deep classifier, four expected outcomes of the confusion matrix are defined as follows: true positive (TP) showed the true diagnosis of chest diseases. True negative (TN) is a measured number of healthy cases. False positive (FP) is a test result incorrectly indicates the presence of a chest disease when the disease is not existing, while a false negative (FN) is the opposite error if the test result fails to indicate the presence of a chest disease. Five basic performance metrics of deep image classifiers are derived from the outcomes of confusion matrix as follows.

**Table 3:** Confusion matrix

	Predicted cases	
	Chest disease	No chest disease
Actual cases		
Positive	True positive (TP)	False negative (FN)
Negative	False positive (FP)	True negative (TN)

*Accuracy* is the most important metric for evaluating the performance of each proposed classifier, as represented in (1). It calculates the number of images correctly classified divided by the total number of X-ray images in the dataset. The precision recall or sensitivity, specificity, and f1-score are also computed by formulae as given below in (2)–(5):

1 $$ {\text{Accuracy}} = \frac{{{\text{TP}} + {\text{TN}}}}{{{\text{TP}} + {\text{FP}} + {\text{FN}} + {\text{TN}}}}, $$

2 $$ {\text{Precision}} = \frac{\text{TP}}{{{\text{TP}} + {\text{FP}}}}, $$

3 $$ {\text{Recall}} = {\text{Sensitivity}} = \frac{\text{TP}}{{{\text{TP}} + {\text{FN}}}}, $$

4 $$ {\text{Specificity}} = \frac{\text{TN}}{{{\text{TN}} + {\text{FP}}}}, $$

5 $$ {\text{f}}1{\text{-score}} = \frac{{2({\text{Precision}} \times {\text{Recall}})}}{{{\text{Precision}} + {\text{Recall}}}}. $$

## Experiments

### Experimental setup

All X-ray images were converted to the RGB format with a fixed size of 150 × 150 pixels. The cascaded deep learning classifiers have been implemented based on a free and open-source Anaconda Navigator with Scientific Python Development Environment (Spyder V3.3.6) including the Keras package with TensorFlow [56] using a PC with Intel(R) Core(TM) i7-2.2 GHz processor. Running deep learning classifiers were done using a graphical processing unit (GPU) of 4 GB NVIDIA GTX1050Ti and RAM of 16 GB.

### Performance evaluation of cascaded classifiers

About 263 X-ray images of the dataset including normal cases, COVID-19 and pneumonia diseases are randomly chosen for training and validation phase. In this study, the proposed CNN models were trained using the hyperparameter values, as illustrated above in Table 2. The number of epochs and batch size are set to 50 and 7, respectively, to accomplish the targeted convergence with few iterations, avoiding the possible degradation problem. All deep neural networks are trained using Stochastic Gradient Descent (SGD) optimizer because of its fast running time and accurate convergence. Data augmentation was exploited to fix imbalanced dataset and avoid overfitting during training [34]. Therefore, the training set of X-ray images was randomly flipped horizontally and vertically, rotated up to ± 15°, and shifted ± 20% of the height and width ranges. Then the total number of X-ray images has been significantly increased to 3325 images for each clinical case to enhance the training performance of proposed deep learning classifiers, as illustrated in Table 4.

**Table 4:** Data augmentation results for training set of chest X-ray images

Patient case	Original images	Augmented images	Total training images
Normal	56	3269	3325
COVID-19	49	3276	3325
Viral (Non-COVID-19) pneumonia	56	3269	3325
Bacterial pneumonia	56	3269	3325

**Fig. 2:**
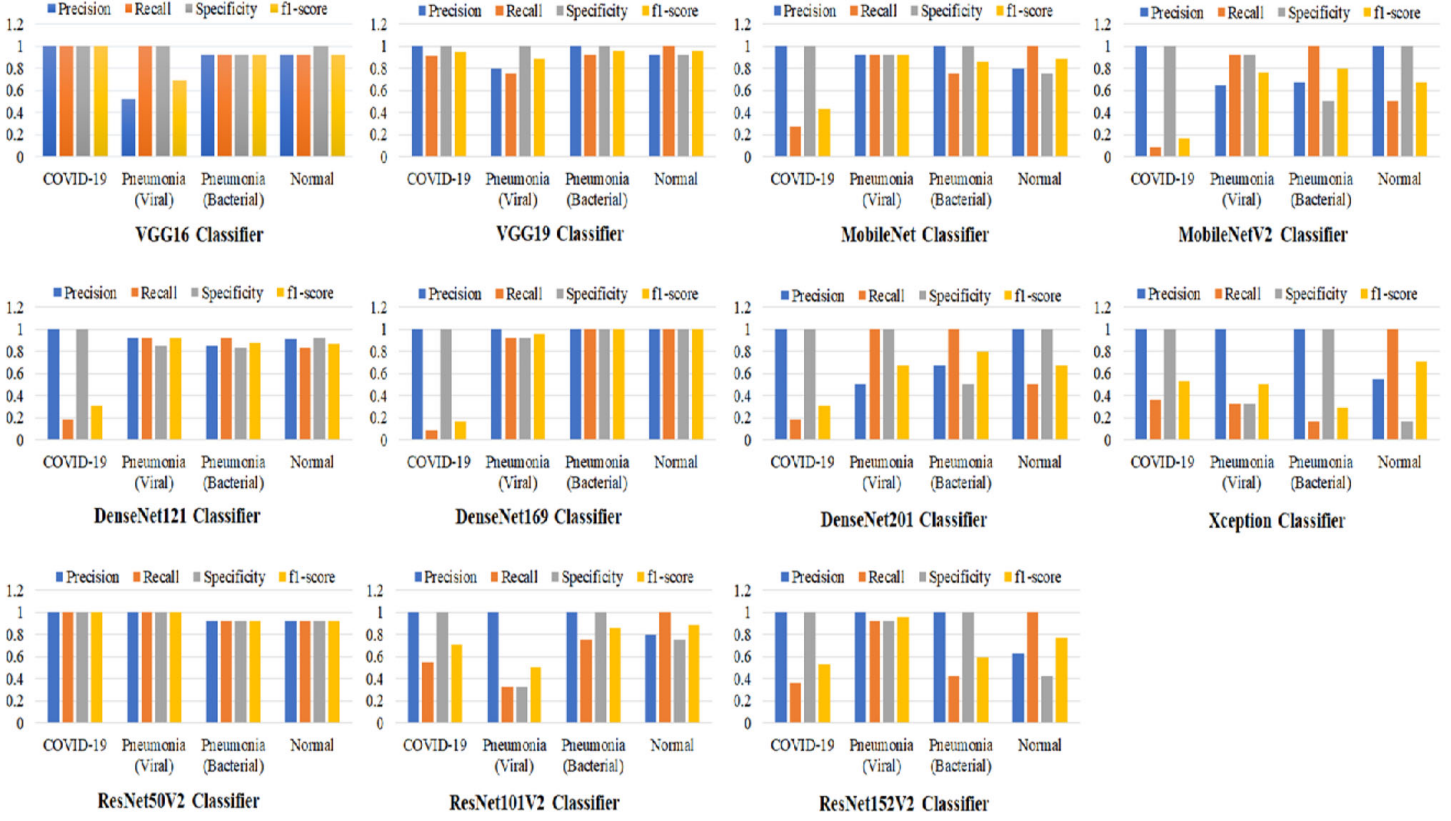
Evaluation results of cascaded deep learning classifiers to identify positive cases of COVID-19, viral and pneumonia diseases. Normal cases indicate healthy patients or other pulmonary diseases not defined in this study

Figure 2 shows the graphical evaluation of all proposed X-ray image classifiers with four metrics of precision, recall, specificity and f1-score as given in (2)–(5). For confirming positive COVID-19 cases, VGG16 and ResNet50V2 achieved the best score of the above metrics, while the misclassification appeared significantly with DensNet169 and MobileNetV2 classifiers. The ResNet101V2 and Xception models could not successfully detect viral pneumonia in tested X-ray images, but the ResNet50V2 classifier showed the best performance to identify this kind of pneumonia infection. Best classification scores of both bacterial pneumonia and normal cases have been achieved by the DenseNet169 classifier.

**Fig. 3:**
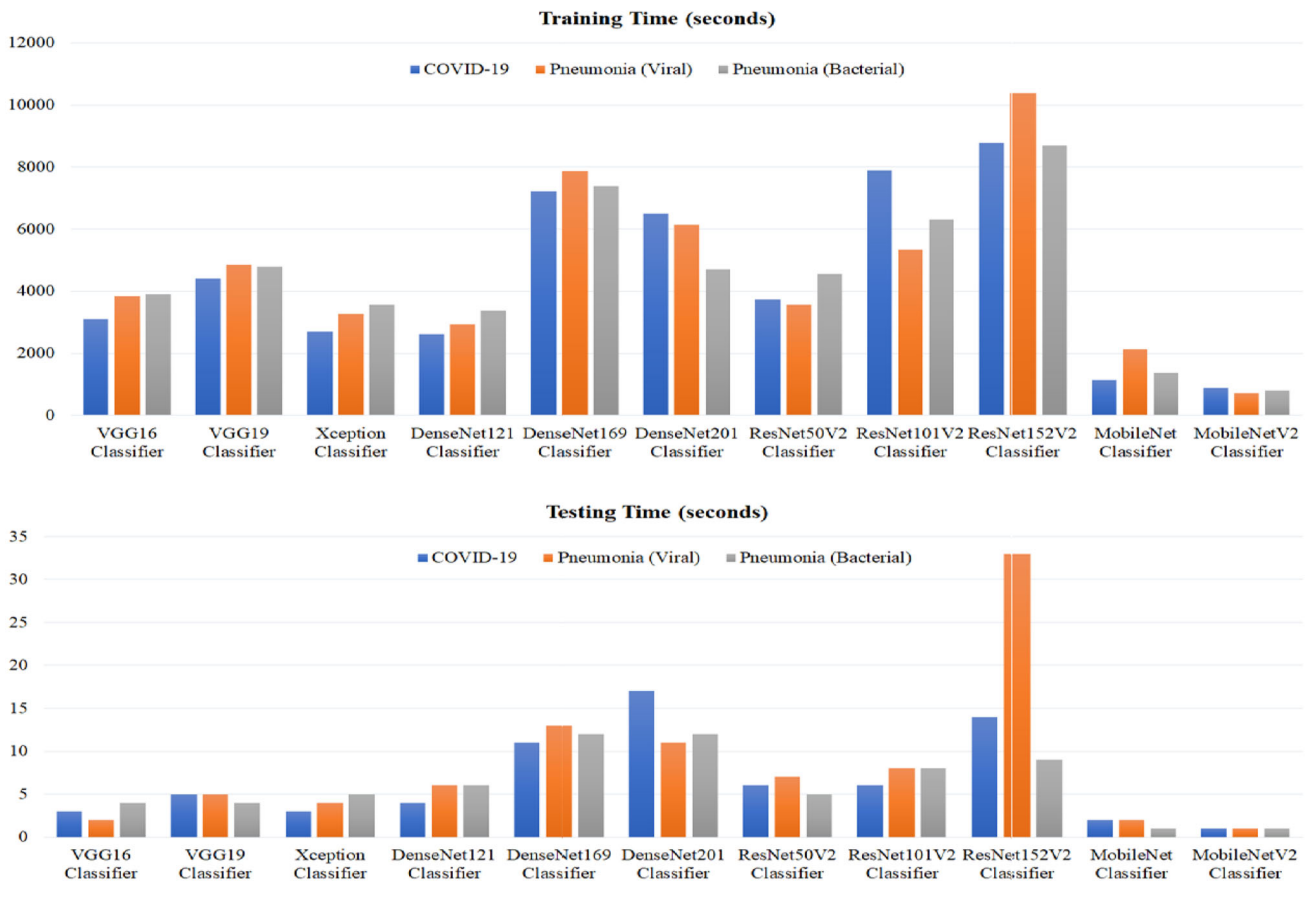
Computation times including training and testing times for all deep learning image classifiers in this study

Comparative computational times including training and testing times (in seconds) of all tested image classifiers are depicted in Fig. 3. The training times of proposed deep learning models are ranging approximately from 12.0 min for the MobileNetV2 classifier to 173.0 min for the ResNet152V2 classifier. The main advantage of using either the MobileNet or the MobileNetV2 classifiers is obviously verified by achieving the shortest computational times in the range of 12.0–35.5 min for the training phase and less than 2 s for testing all cases. The testing times of deep learning models did not exceed 33.0 s (for the ResNet152V2 classifier), as shown in Fig. 3.

**Fig. 4:**
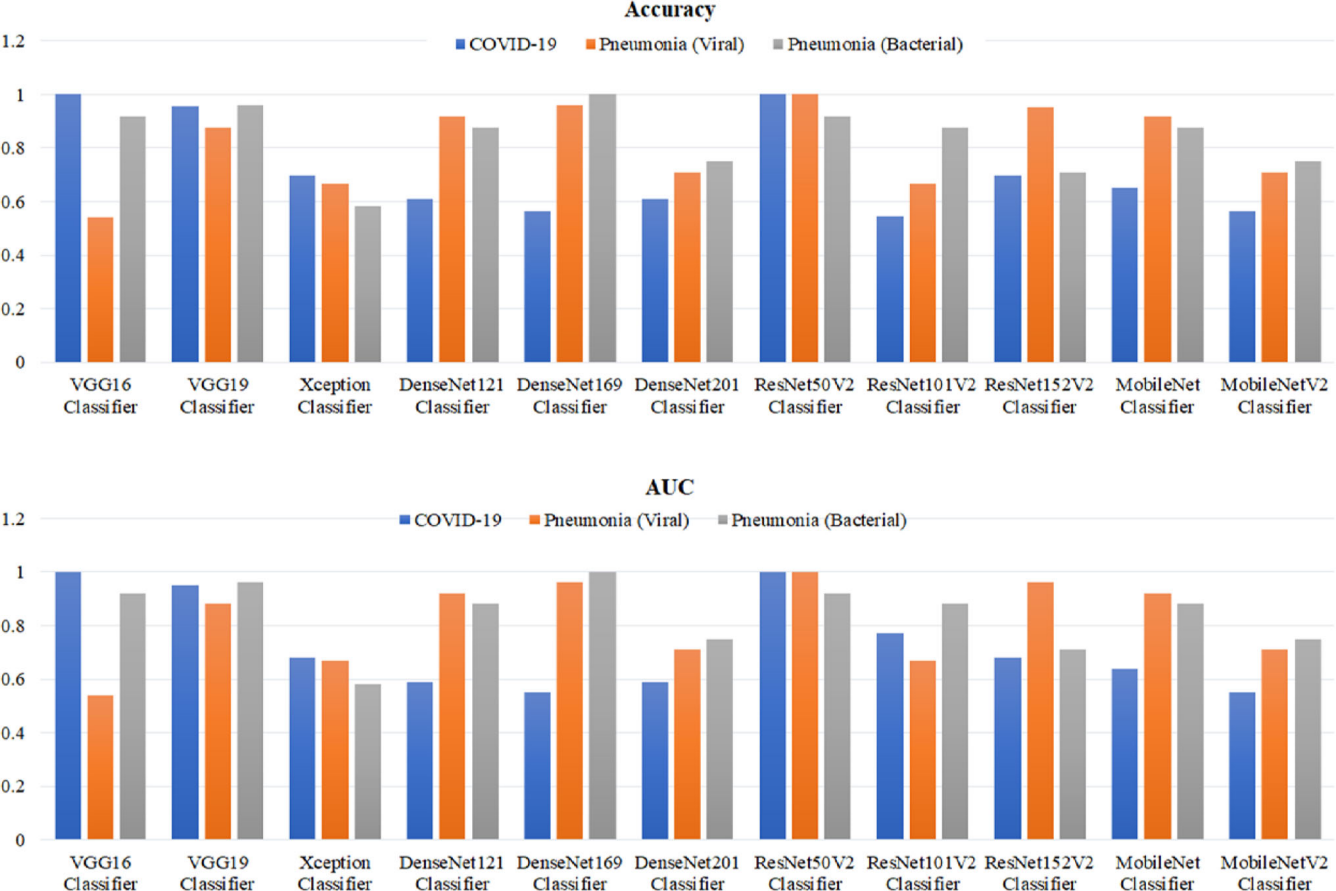
Classification accuracy and area under curve (AUC) for all cascaded deep learning classifiers in this study

Figure 4 shows the comparative testing accuracy and the values of area under curve (AUC) for all tested cascaded image classifiers. The best scores of both accuracy and AUC have been achieved by the ResNet50V2 above 90% for all diseased and normal cases. In contrast, the Xception classifier resulted worst scores of the accuracy and AUC less than 70%. The MobileNet and MobileNetV2 classifiers showed average values of 55.0–92.0% for the classification accuracy and AUC, but these models can be re-optimized to improve their performance, considering their efficient computational times for confirming the COVID-19 infection using smart devices.

Moreover, Table 5 gives another comparison between the characteristics of each deployed deep learning model and the corresponding classification accuracy for all tested cases. Although the MobileNetV2 model has the lowest total number of parameters approximately 2.34 × 10^6^, it could not be trained well to handle the features of chest X-ray images. Consequently, the classification accuracy did not exceed 75% for the MobileNetV2 classifier. On the other side, the VGG16 and VGG19 models are fully trained and can achieve a good accuracy for two cases of COVID-19 and bacterial pneumonia above 90.0%, but they failed to detect viral pneumonia disease correctly. The highest total number of network parameters is recorded about 58.5 × 10^6^ for the ResNet152V2 model, which could not classify bacterial pneumonia disease accurately. The ResNet50V2 model with minimal non-trainable parameters achieved the most accurate classification results for COVID-19 and viral pneumonia diseases. The DenseNet169 has relatively high number of non-trainable parameters about 0.16 × 10^6^, but it can identify the case of bacterial pneumonia disease successfully, as illustrated in Table 5.

**Table 5:** Comparative characteristics of deployed deep learning models to the resulted classification accuracy

Classifier	Total parameters (10^6^)	Trainable parameters (10^6^)	Non-trainable parameters (10^6^)	Classification accuracy (%)		
				COVID-19	Viral pneumonia	Bacterial pneumonia
**VGG16**	14.75	14.75	**0.00**	**99.90**^a^	54.17	91.67
VGG19	20.06	20.06	**0.00**	95.65	87.50	95.83
Xception	20.99	20.94	0.05	69.57	66.67	58.33
DenseNet121	7.10	7.02	0.08	91.67	91.67	87.50
**DenseNet169**	12.75	12.59	0.16	95.83	95.83	**99.90**
DenseNet201	18.45	18.22	0.23	70.83	70.83	75.00
**ResNet50V2**	23.70	23.65	0.05	**99.90**	**99.90**	91.67
ResNet101V2	42.76	42.66	0.10	66.67	66.67	87.50
ResNet152V2	58.46	58.32	0.14	95.00	95.00	70.83
MobileNet	3.29	3.27	0.02	91.67	91.67	87.50
MobileNetV2	2.34	2.31	0.03	70.83	70.83	75.00

^a^The bold value indicates the best performance

Furthermore, comparative accuracy scores of previous methods in the literature and our proposed cascaded classifiers are reported in Table 6. These deep learning classifiers have been evaluated on the same X-ray dataset in [47]. It is obvious that our proposed method achieved the best accuracy of 99.9% for multi-label classification of COVID-19 and pneumonia diseases, outperforming other methods in previous studies. The concatenation of different deep learning models in a cascaded form presents a powerful and unique advantage in this study, because the user can select the best performance of deep learning model to classify each diseased case separately instead of one complex classifier for all pulmonary diseases.

**Table 6:** Comparison of the proposed method with other deep learning methods in previous studies

Authors	Deep learning classifier	Diseases	Accuracy (%)
Hemdan et al. [36]	COVIDX-Net	COVID-19	90.0
Narin et al. [37]	ResNet50 InceptionV3 Inception-ResNetV2	COVID-19	98.0 97.0 87.0
Apostolopoulos and Mpesiana [38]	Transfer learning with CNN models	COVID-19 Common pneumonia	93.48
Wang and Wong [39]	COVID-Net	COVID-19 Non-COVID-19 pneumonia	92.4
Ghoshal and Tucker [40]	Bayesian CNN	COVID-19 Non-COVID-19 viral Bacterial pneumonia	Almost 90.0 (combined with an experienced radiologist)
Our proposed method	Cascaded classifiers (VGG16, ResNet50V2, DenseNet169)	COVID-19 Non-COVID-19 viral Bacterial pneumonia	99.9

### Final recommended image classifiers

**Fig. 5:**
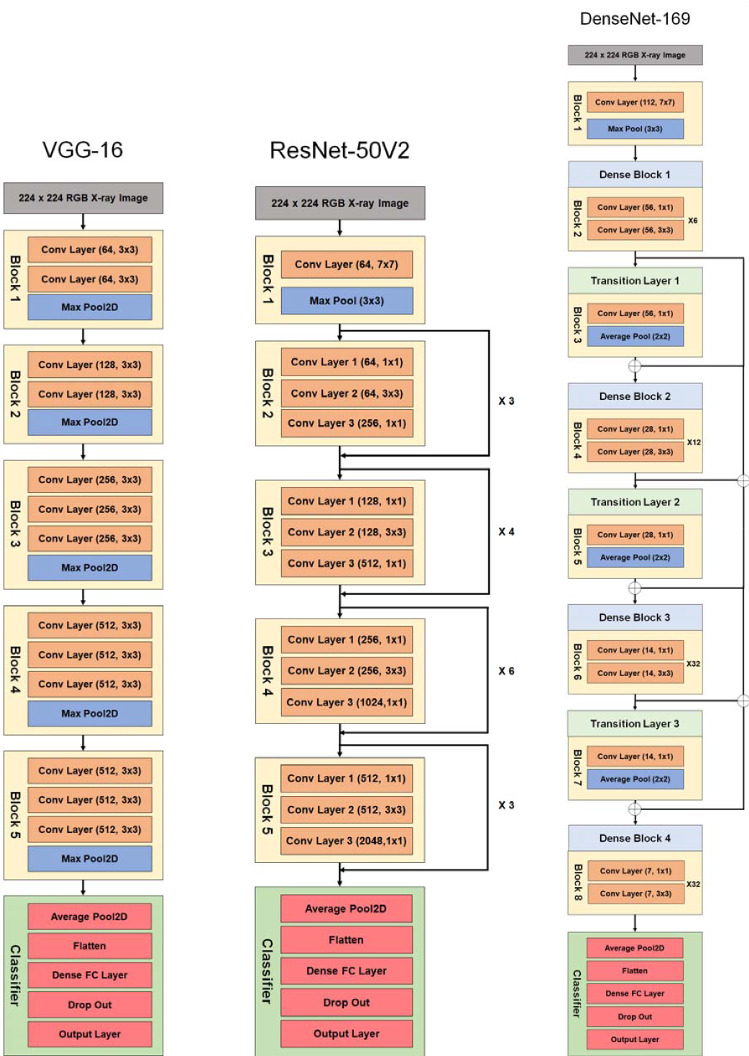
Architectures of the best fine-tuning models, namely VGG16, ResNet50V2, and DenseNet169 for detecting COVID-19, viral and bacterial pneumonia diseases in X-ray images, respectively

**Fig. 6:**
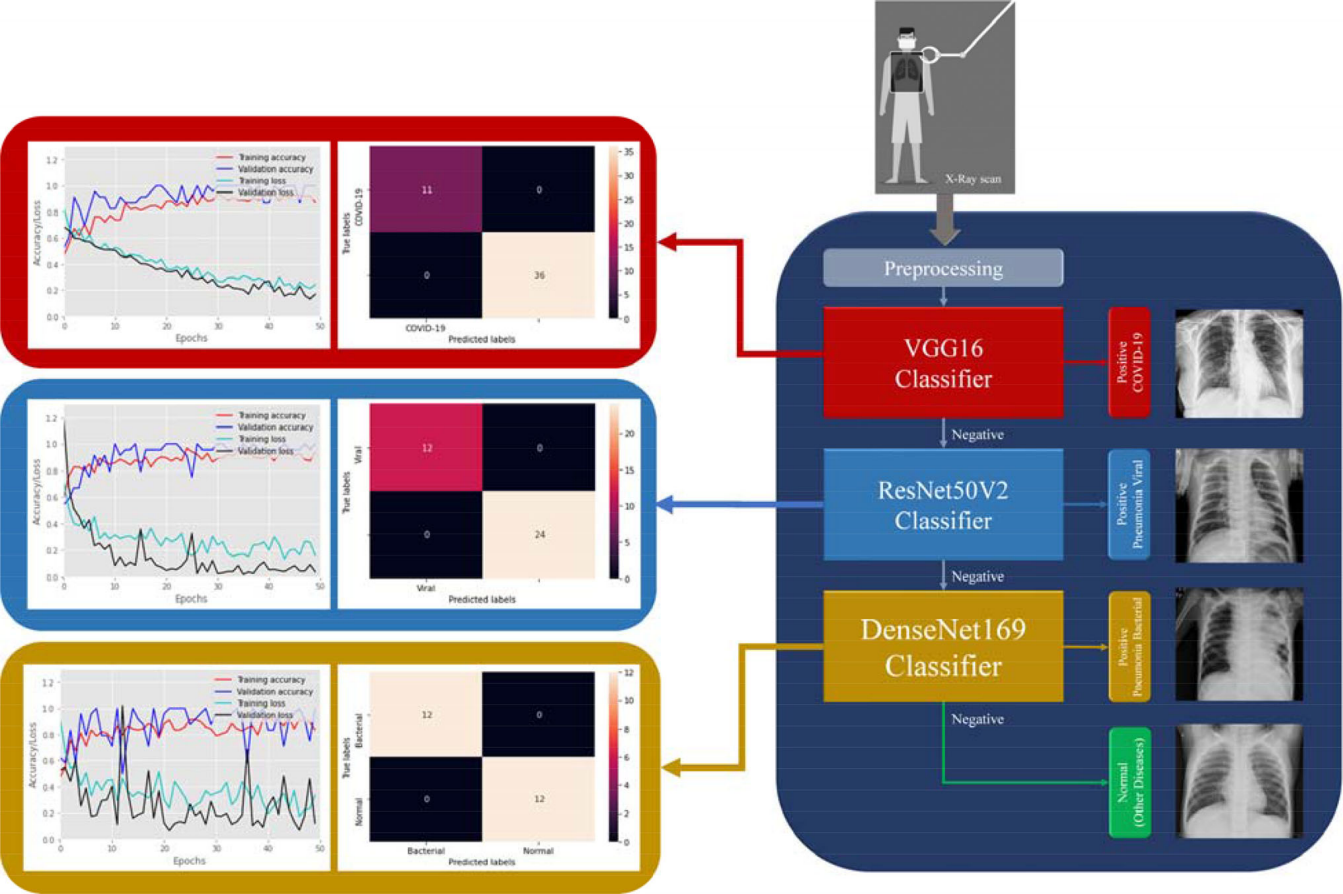
Final recommended deep X-ray image classifiers for detecting COVID-19, viral and bacterial pneumonia diseases using VGG16, ReseNet50V2 and DenseNet169 models, respectively (right). The corresponding accuracy and loss curves with resulted confusion matrix are linked to each image classifier (left)

The above evaluation results demonstrated that our proposed deep learning framework can be finalized in multi-stage X-ray image classification, as depicted in Figs. 5 and 6. The VGG16 and ResNet50V2 models showed the best performance and classification accuracy of 99.9% to identify COVID-19 cases. However, the VGG16 classifier is faster than the ResNet50V2 classifier for training and testing times, as shown in Fig. 3. Also, the structure of VGG16 is simpler than the ResNet50V2 model, as summarized in Table 5. Therefore, it is selected as the first stage of classification procedure for detecting Coronavirus infection in X-ray images. In the second stage of viral (Non-COVID-19) pneumonia classification, the ResNetV2 model achieved the best performance (see Fig. 4). Finally, the DenseNet169 model is selected to detect the bacterial pneumonia disease because of its superior performance to other deep learning models as reported in Table 5. Also, Fig. 6 shows the resulted confusion matrix and the accuracy/loss curves to ensure the outstanding performance of our recommended deep image classifiers in this study.

Developing a generic X-ray and/or CT image classifier is still challenging to assist radiologists during the diagnostic procedures of COVID-19 and pulmonary diseases. Many previous studies proposed effective deep learning models to perform this crucial task using many image datasets, but they did not suggest a practical option for the manual supervision of clinicians to customize these classifiers according to their clinical environment. Our proposed framework provides this manual option to get recommended deep learning classifiers as shown in Figs. 1 and 6. It can be easily re-trained on new image dataset, adding or selecting other cascaded deep learning models to precisely identify COVID-19 infections and pulmonary diseases in chest X-ray images.

## Conclusions and outlook

This study presented a new framework for automated computer-aided diagnosis of COVID-19, viral and bacterial pneumonia in chest X-rays, based on three cascaded deep learning classifiers. Compared to previous studies, the proposed cascaded classifiers achieved promising results to confirm positive COVID-19 cases using VGG16 model. Also, the ResNet50V2 and DenseNet169 models identified viral and bacterial diseases successfully, as shown above in Fig. 6. Hence, we are currently working on the clinical implantation of our deep learning framework as a new CAD system in the diagnostic protocol of potential COVID-19 patients with other pneumonia diseases using the cost-effective X-ray imaging modality.

Furthermore, automated segmentation of COVID-19 infections in chest X-ray scans is the main prospect of this research work. This segmentation task will significantly assist the clinician to follow-up the disease progress in the lung of infected patients, as described in [57]. To satisfy security and privacy requirements for transmitting medical images over general communication networks [58], securing COVID-19 patient data will be also considered in the next version of our developed deep learning framework.
